# “Brain-Doping,” Is It a Real Threat?

**DOI:** 10.3389/fphys.2019.00483

**Published:** 2019-04-24

**Authors:** Darías Holgado, Miguel A. Vadillo, Daniel Sanabria

**Affiliations:** ^1^Department of Physical Education and Sport, Faculty of Sport Sciences, University of Granada, Granada, Spain; ^2^Department of Experimental Psychology, Mind, Brain and Behavior Research Centre, University of Granada, Granada, Spain; ^3^Department of Basic Psychology, Autonomous University of Madrid, Madrid, Spain

**Keywords:** brain stimulation, exercise performance, doping, tDCS — transcranial direct current stimulation, meta-analaysis, sports performance

Since the term “Neurodoping” was introduced (Davis, 2013; Reardon, 2016), the use of transcranial direct current stimulation (tDCS) has gained popularity in Sports Science within a short space of time, based on the same straightforward logic: if exercise is to some extent determined by brain activity, then stimulating brain areas related to exercise should improve physical and sport performance. In fact, companies like Halo Sport claim that their “do-it-yourself” tDCS device has ergogenic effects and can increase sport and exercise performance (Reardon, 2016). In a recent review in Frontiers in Physiology, Angius et al. (2017) suggested that tDCS might have a positive effect on exercise capacity, although the mechanisms of that potential benefit were unknown. However, the expectations derived from those initial studies showing tDCS as an effective technique to increase exercise performance or reduce rate of perceived exertion (RPE), have left room for many others that do not seem to support the effectiveness of tDCS in the Sports science.

Indeed, recent meta-analyses have challenged the idea that tDCS can increase exercise performance or reduce RPE, mood or pain related to exercise (Holgado et al., 2018; Lattari et al., 2018a; Machado et al., 2018). For instance, based on the analysis of 36 effect sizes, Holgado et al. (2018)showed that the effect (if any) of tDCS on exercise performance is rather small (*g* = 0.34) and possibly inflated by methodological artifacts and selective publication. Similarly, the results of Machado et al. (2018) support the conclusion that tDCS has no effect on measures of muscle strength, although it may have a positive effect on cycling exercise. However, even this positive result seems to be entirely dependent on a single, low-quality study. Therefore, both Holgado et al. (2018) and Machado et al. (2018) reached the same conclusion: tDCS has little or no-effect on exercise performance. Moreover, it is worth mentioning that the chances of a publication bias in this topic are particularly high, that is, many other studies with null findings may not have been published or even sent for review (Holgado et al., 2018). So far, only one meta-analysis (Lattari et al., 2018a) has concluded that tDCS may be useful to increase performance. However, upon closer inspection, these results also seem to be grossly influenced by individual studies with unusually large effect sizes (*g* = 3.56 for Cogiamanian et al., 2007 and *g* = 1.94 for Lattari et al., 2018b), casting doubts on the reliability of these effects.

The site of stimulation (Table 1), the fitness level of participants, the overly low sample sizes (average *N* = 15; which may lead to overestimation of effect sizes) and the likely ineffectiveness of the usual stimulation intensity (1–2 mA; cf. Vöröslakos et al., 2018) are key issues that would need to be considered in future research. Regarding stimulation intensity, recent studies have proposed that due to the high inter-individual variability in participants' electric fields, it seems that the most efficient approach to induce constable cortical changes would be to apply an individualized current intensity for each person (Esmaeilpour et al., 2018; Laakso et al., 2019). Indeed, the usual stimulation intensity does not seem to induce oscillatory brain electrical changes at rest or during exercise (e.g., Holgado et al., 2019; cf. Vöröslakos et al., 2018). Finally, the sham procedure might provide an additional source of variability, since without the appropriate procedure sham stimulation might have biological effects (Fonteneau et al., 2019).

**Table 1:** The most common placement of the tDCS' electrodes in the Sport Science research and its rationale.

**Electrode placement**	**Rationale**
Primary motor cortex (M1)	Increase M1 excitability to speed neural drive to active muscles Modulate pain perception
Prefrontal cortex	Changes in pacing behavior via executive functions, for example, by increasing inhibitory control capacity
Temporal cortex	Modulate the activity of the insular cortex Autonomic cardiac control Changes in self-perception and awareness of body sensations
Supplementary motor area	Reduce perceived exertion during exercise

In our opinion, the current evidence does not support the effectiveness of tDCS devices in the sport domain. It is therefore premature to make claims regarding the ergogenic benefits of tDCS and/or its potential thread as a novel doping tool. We believe, however, that this line of scientific enquiry could provide valuable knowledge if researchers endorse sound scientific practices (e.g., pre-registration, testing larger sample, multi-lab replications, etc.), to tackle issues like the role of stimulation intensity, the site of stimulation, and the inter-individual variability.

## Author Contributions

All authors listed have made a substantial, direct and intellectual contribution to the work, and approved it for publication.

### Conflict of Interest Statement

The authors declare that the research was conducted in the absence of any commercial or financial relationships that could be construed as a potential conflict of interest.
